# Intravascular large B-cell lymphoma: presentation with generalized dendritic-type telangiectasias. A diagnostic challenge^؆^

**DOI:** 10.1016/j.abd.2025.501221

**Published:** 2025-11-06

**Authors:** Catalina Aitken, Alfonso Lépez, Esteban Araos-Baeriswyl, Mauricio Lechuga, Claudio Pinto, Isabel Henríquez

**Affiliations:** a Department of Dermatology, School of Medicine, Pontificia Universidad Católica de Chile, Santiago, Chile; b Dermatology Service, Hospital Dr. Sótero del Río, Puente Alto, Santiago, Chile; c Department of Pathology, Hospital Dr. Sótero del Río, Puente Alto, Santiago, Chile

Dear Editor,

Intravascular lymphoma is an extremely rare disease with diverse and atypical clinical manifestations, making early diagnosis challenging and impacting the prognosis.^1^

We present the case of a 70-year-old female patient with a history of cervical cancer treated with surgery, brachytherapy, and radiotherapy, who presented with a two-year history of progressive edema in both lower extremities, associated with multiple generalized telangiectasias (Fig. 1), significant weight loss, episodes of fever up to 38 °C, and night sweats. Laboratory tests revealed hemoglobin of 8.7 g/dL, MCV 94 fL, leukocytes 3,240 μL with neutrophil predominance, platelets 144,000 μL, ESR 48 mm/h, uric acid 6.2 mg/dL, LDH 662 U/L (RI: 125‒220), ALP 246 U/L (RI: 40‒150), GGT 135 U/L (RI: 9‒36), normal transaminases and bilirubin levels, and vitamin B12 at 1360 pg/mL, with normal renal function, calcium, and phosphorus levels. β2-microglobulin was elevated (3.74 mg/L). Protein electrophoresis showed a monoclonal peak in the gamma region, with an IgM kappa monoclonal component and lambda light chain on immunofixation. Computed tomography (CT) of the chest, abdomen, and pelvis showed no significant pathological findings. Doppler ultrasound of the lower extremities revealed subcutaneous edema. Bone marrow biopsy was normal. A skin biopsy was performed, identifying large atypical lymphoid cells with prominent nucleoli within small vessels (Fig. 2). Immunohistochemistry was positive for CD20, PAX-5, MUM-1, and Bcl-2 and negative for CD3, Bcl-6, CD10, CD56, CD30, CD34, and cytokeratins (Fig. 3). A diagnosis of intravascular large B-cell lymphoma (IVLBCL) was established. The patient was initiated on the R-CHOP chemotherapy regimen (rituximab, cyclophosphamide, doxorubicin, vincristine, and prednisone). A marked reduction in cutaneous telangiectasias ‒ estimated at approximately 60% ‒ was noted following the first cycle. Intrathecal methotrexate was also administered due to the presence of pathological mononuclear cells in moderate quantity in the cerebrospinal fluid, which subsequently cleared. The patient remains under multidisciplinary follow-up by hematology and dermatology, exhibiting a favorable clinical response after six cycles of R-CHOP, with only sparse residual telangiectasias noted on the chest.

**Figure 1:**
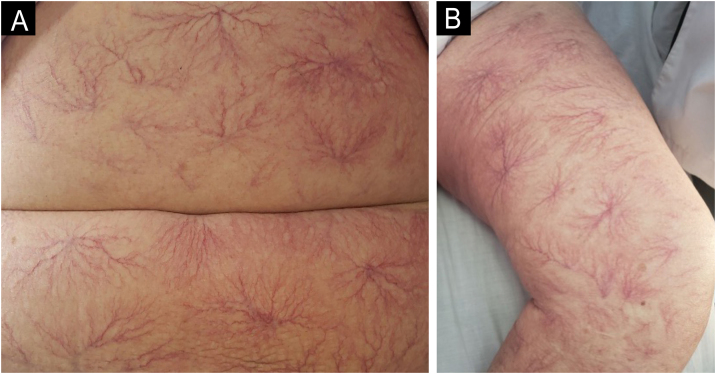
Clinical images. Multiple generalized dendritic type telangiectasias on the abdomen (A) and thigh (B).

**Figure 2:**
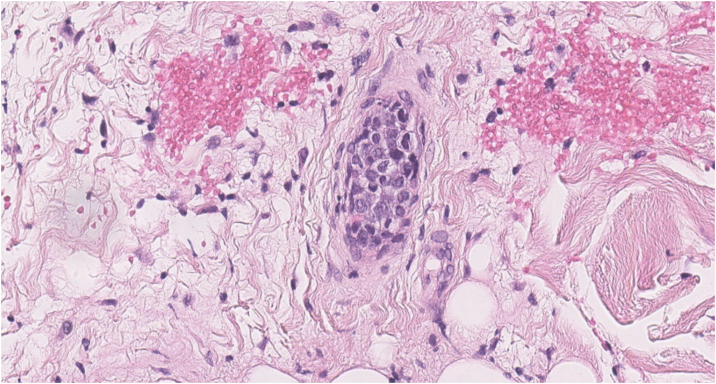
Atypical intravascular lymphoid population with monomorphic, uniformly enlarged cells with prominent nucleoli (Hematoxylin & eosin, ×400).

**Figure 3:**
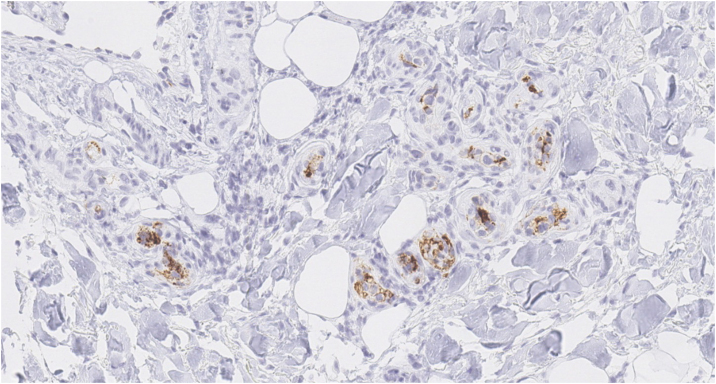
Immunohistochemistry showing hyperchromatic cells showing positive immunohistochemical staining with the CD20 B-cell marker (×400).

Intravascular lymphoma is a rare type of extranodal non-Hodgkin lymphoma (<1% of cutaneous lymphomas), characterized by the proliferation of neoplastic lymphocytes in the lumen of small vessels, described in 1959 as “endotheliomatosis and reticuloendotheliosis”.^2^ An incidence of 0.09 per 1 million inhabitants was reported in the USA between 2000‒2013.^3^ The average age of presentation is 70-years, with no gender differences.^2^, ^3^ 85% originate from B-cells (IVLBCL) and 15% from NK or T-cells.^4^ Two clinical variants are described: the “Asian” variant, associated with multi-organ involvement and hemophagocytosis, and the “Western” variant, associated with skin lesions and central nervous system involvement.^5^, ^6^ Clinical presentation is variable, including the presence of B-symptoms (55%‒85%) without associated lymphadenopathy, neurological symptoms (39%‒76%), and skin lesions (17%‒39%) with erythematous-violet indurated plaques and nodules that simulate panniculitis, associated with edema. Its presentation as dendritic-type telangiectasias is very rare, with only 24 reported cases.^1^, ^2^, ^3^, ^4^, ^5^, ^6^

Due to its variable and nonspecific symptoms, the diagnosis tends to be delayed, leading to a poor prognosis, with a median survival of 46.1% at 5-years.^3^ In cases of timely treatment with R-CHOP, the survival rate is 66% at 24-months, making it a potentially curable disease with chemotherapy.^3^, ^5^ If there is CNS involvement, methotrexate or cytarabine is recommended.^5^ In certain cases, an autologous stem cell transplant should be considered.^2^, ^5^

## ORCID IDs

Alfonso Lépez: 0009-0006-8363-5221

Esteban Araos-Baeriswyl: 0000-0002-5740-9558

Mauricio Lechuga: 0009-0009-7754-7833

Claudio Pinto: 0000-0002-0750-8232

Isabel Henríquez: 0009-0005-6798-1290

## Financial support

None declared.

## Authors' contributions

Catalina Aitken: The study concept and design; writing of the manuscript or critical review of important intellectual content; critical review of the literature; final approval of the final version of the manuscript.

Alfonso Lépez: The study concept and design; writing of the manuscript or critical review of important intellectual content; critical review of the literature; final approval of the final version of the manuscript.

Esteban Araos-Baeriswyl: The study concept and design; writing of the manuscript or critical review of important intellectual content; critical review of the literature; final approval of the final version of the manuscript.

Mauricio Lechuga: The study concept and design; effective participation in the research guidance; intellectual participation in the propaedeutic and/or therapeutic conduct of the studied cases.

Claudio Pinto: Effective participation in the research guidance; intellectual participation in the propaedeutic and/or therapeutic conduct of the studied cases.

Isabel Henríquez: Effective participation in the research guidance; intellectual participation in the propaedeutic and/or therapeutic conduct of the studied cases.

## Research data availability

Does not apply.

## Conflicts of interest

None declared.

## Editor

Síio Alencar Marques.

## References

[1] Cheng J.W., Li J.H. (2023). Intravascular large B-cell lymphoma. N Engl J Med..

[2] Vásquez J., Romero V., Vilas P., Serra-Rexach J.A., Vidán M.T. (2019). Progressive edemas and generalized telangiectasia: a presentation of intravascular B-cell lymphoma. Clin Case Rep..

[3] Rajyaguru D.J., Bhaskar C., Borgert A.J., Smith A., Parsons B. (2017). Intravascular large B-cell lymphoma in the United States (US): a population-based study using surveillance, epidemiology, and end results program and national cancer database. Leuk Lymphoma..

[4] Zhang W., Tang Y. (2024). Generalized telangiectasia as a hallmark of intravascular lymphoma: a case report and literature review. Healthcare (Basel)..

[5] Ponzoni M., Campo E., Nakamura S. (2018). Intravascular large B-cell lymphoma: a chameleon with multiple faces and many masks. Blood..

[6] Koizumi S., Togawa Y., Saeki Y., Shimizu R., Nakano M. (2023). A case of cutaneous variant of intravascular large B-cell lymphoma in which dermoscopy revealed telangiectasias associated with erythematous induration. Dermatol Reports..

