# Supplementary figures and images for: Intravascular large B-cell lymphoma: presentation with generalized dendritic-type telangiectasias. A diagnostic challenge
Source: An Bras Dermatol. 2025 Nov 6;100(6):501221. doi: 10.1016/j.abd.2025.501221 (PMC12683154; doi:10.1016/j.abd.2025.501221)

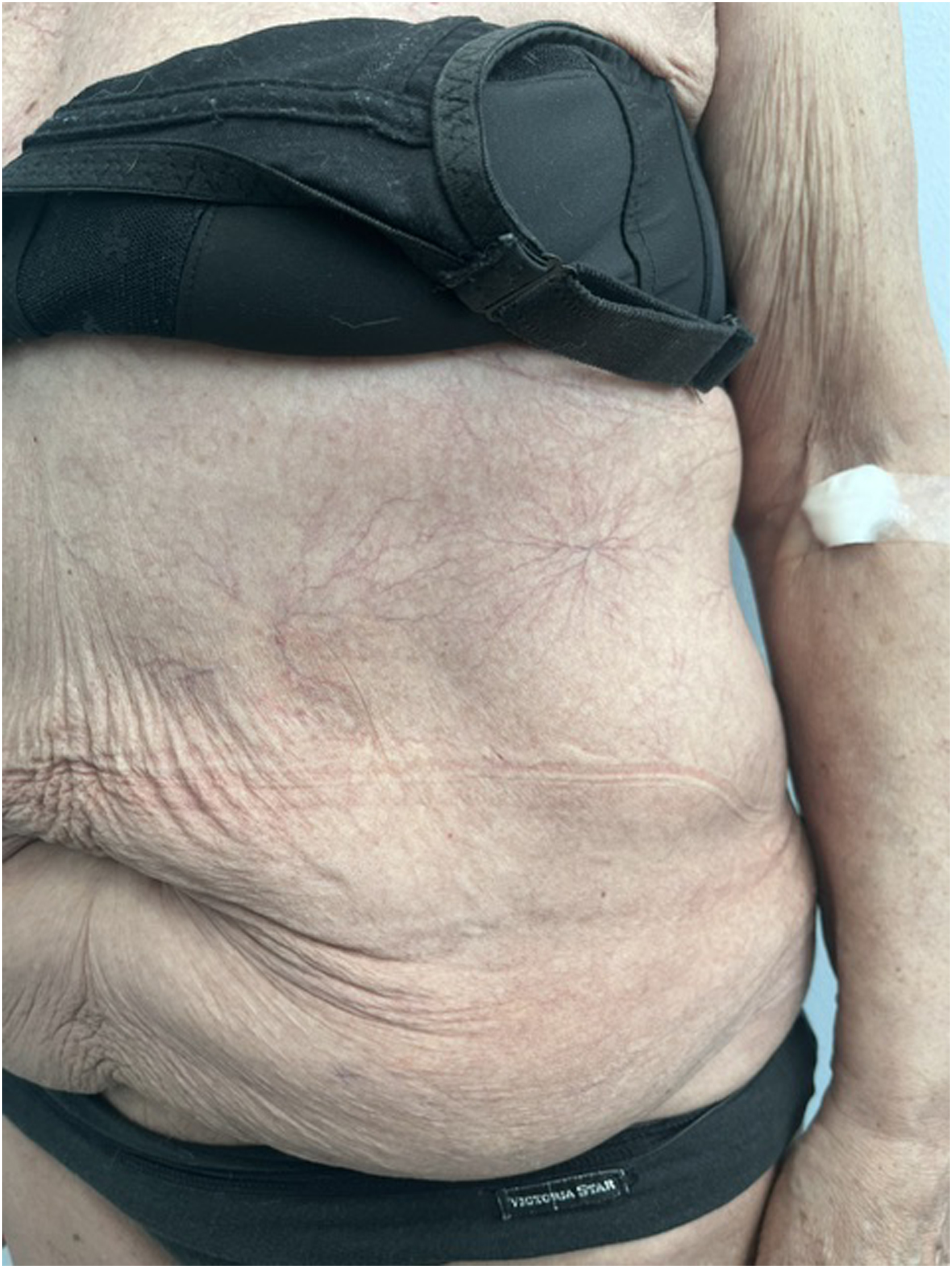

Supplement: Supplementary file 1 [file mmc1.jpg]

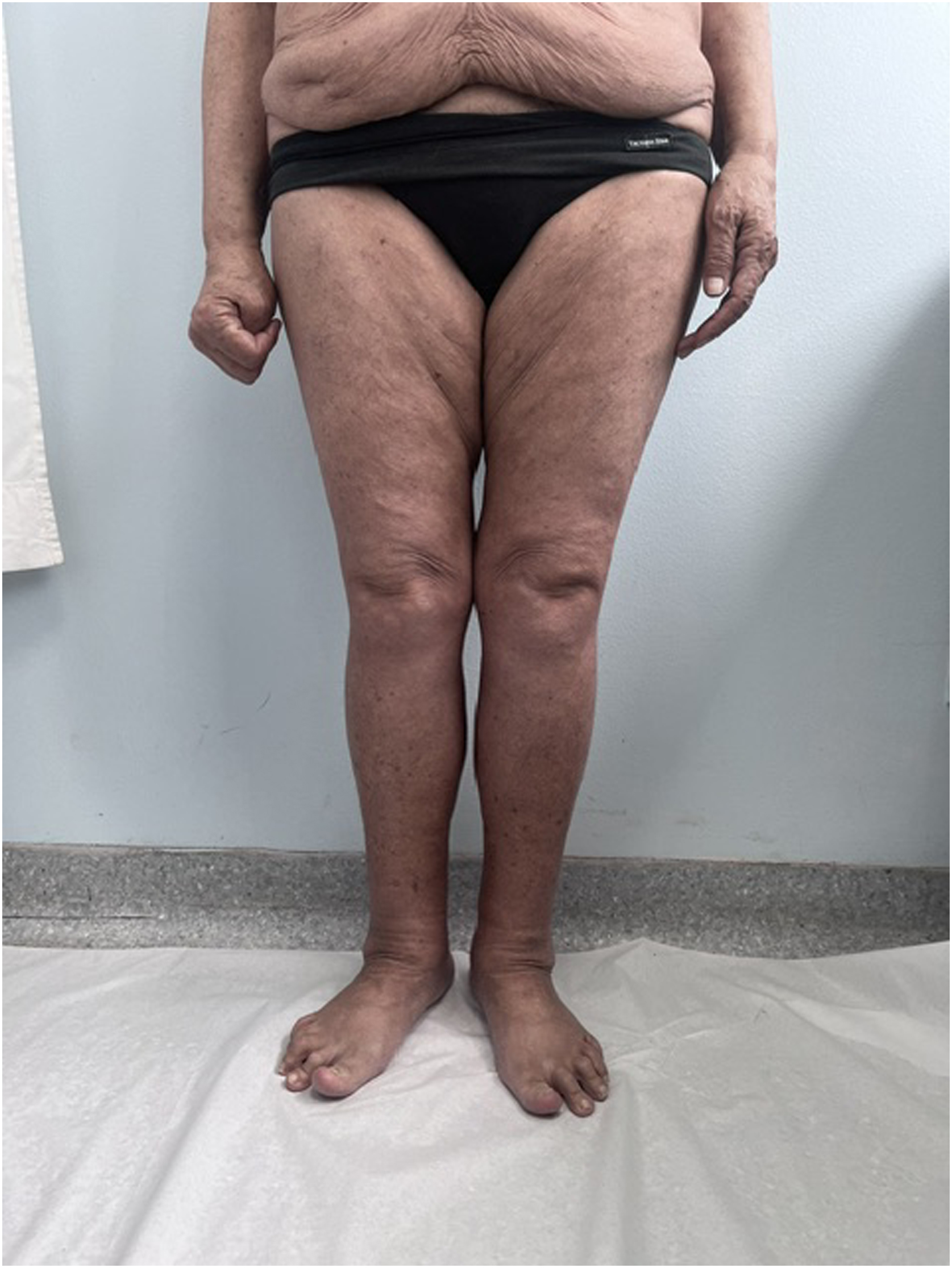

Supplement: Supplementary file 2 [file mmc2.jpg]

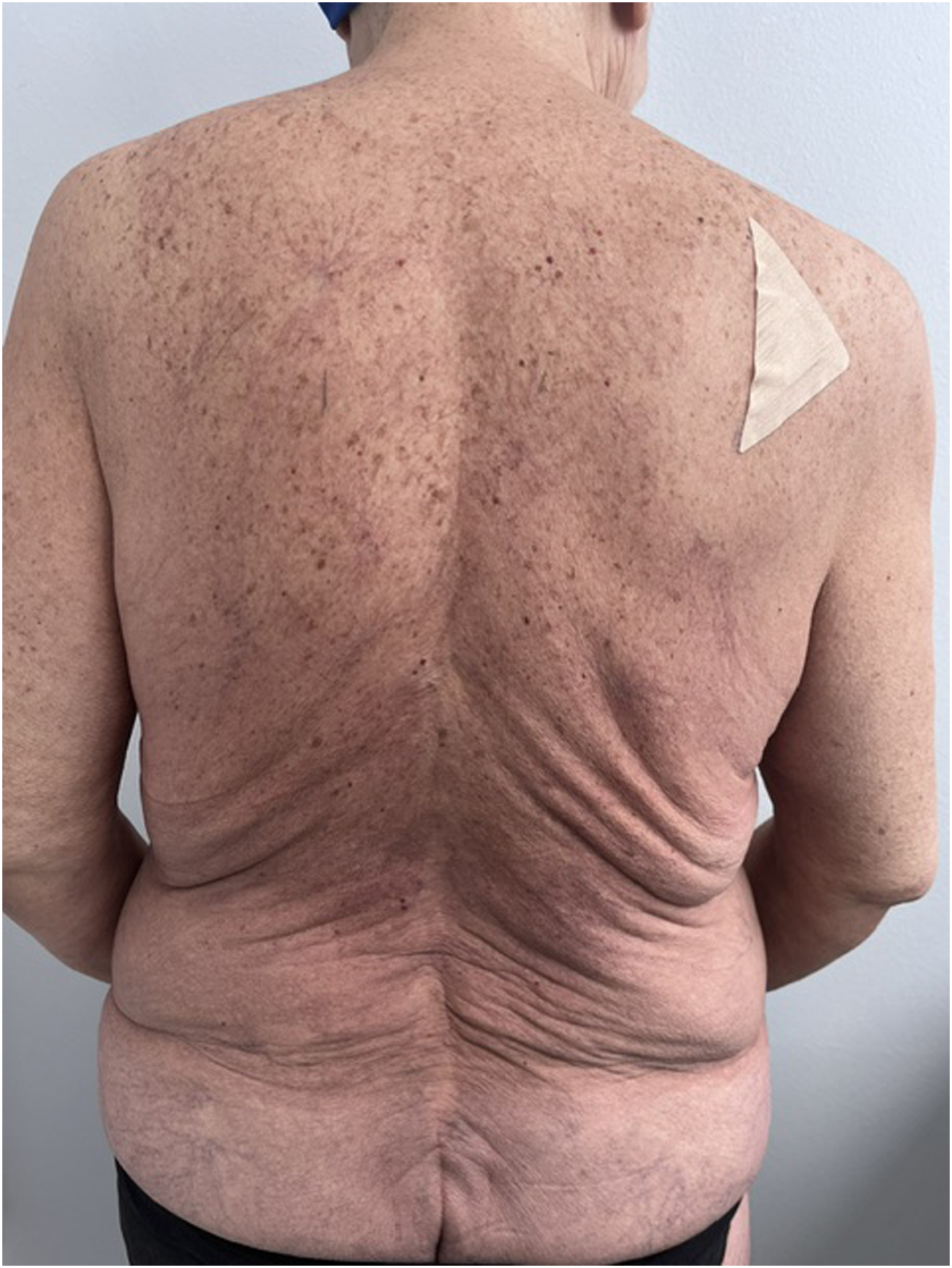

Supplement: Supplementary file 3 [file mmc3.jpg]

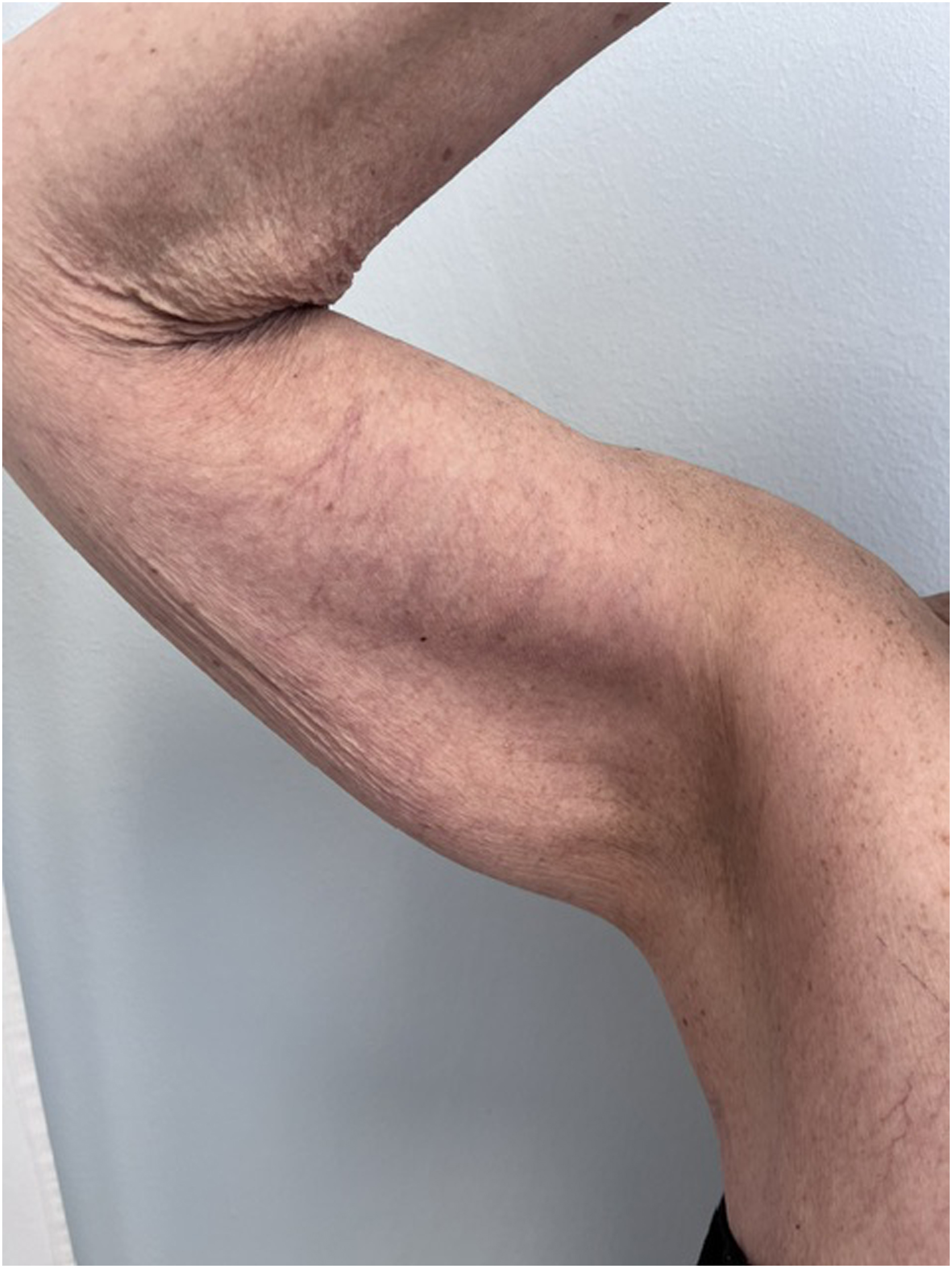

Supplement: Supplementary file 4 [file mmc4.jpg]

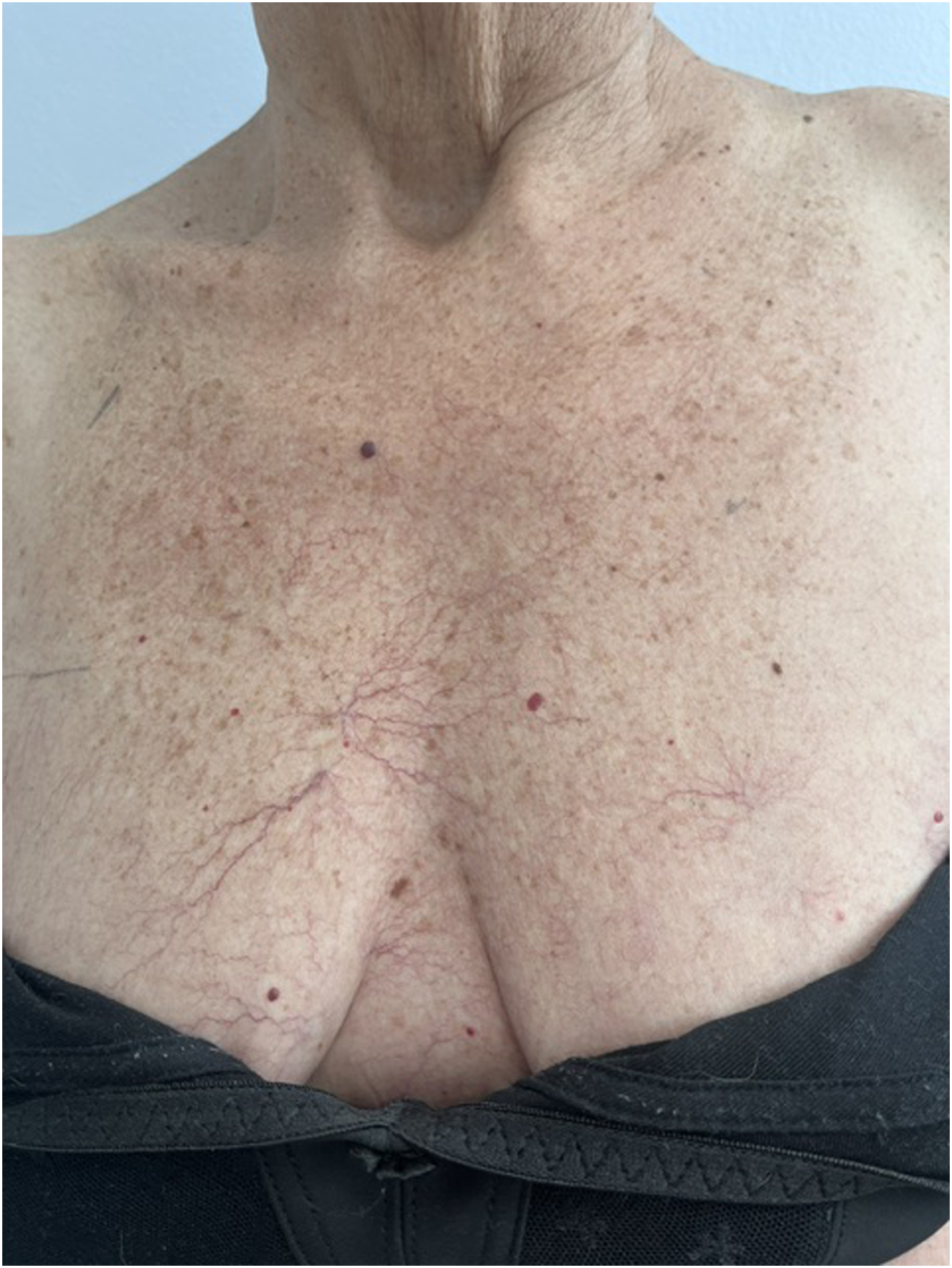

Supplement: Supplementary file 5 [file mmc5.jpg]

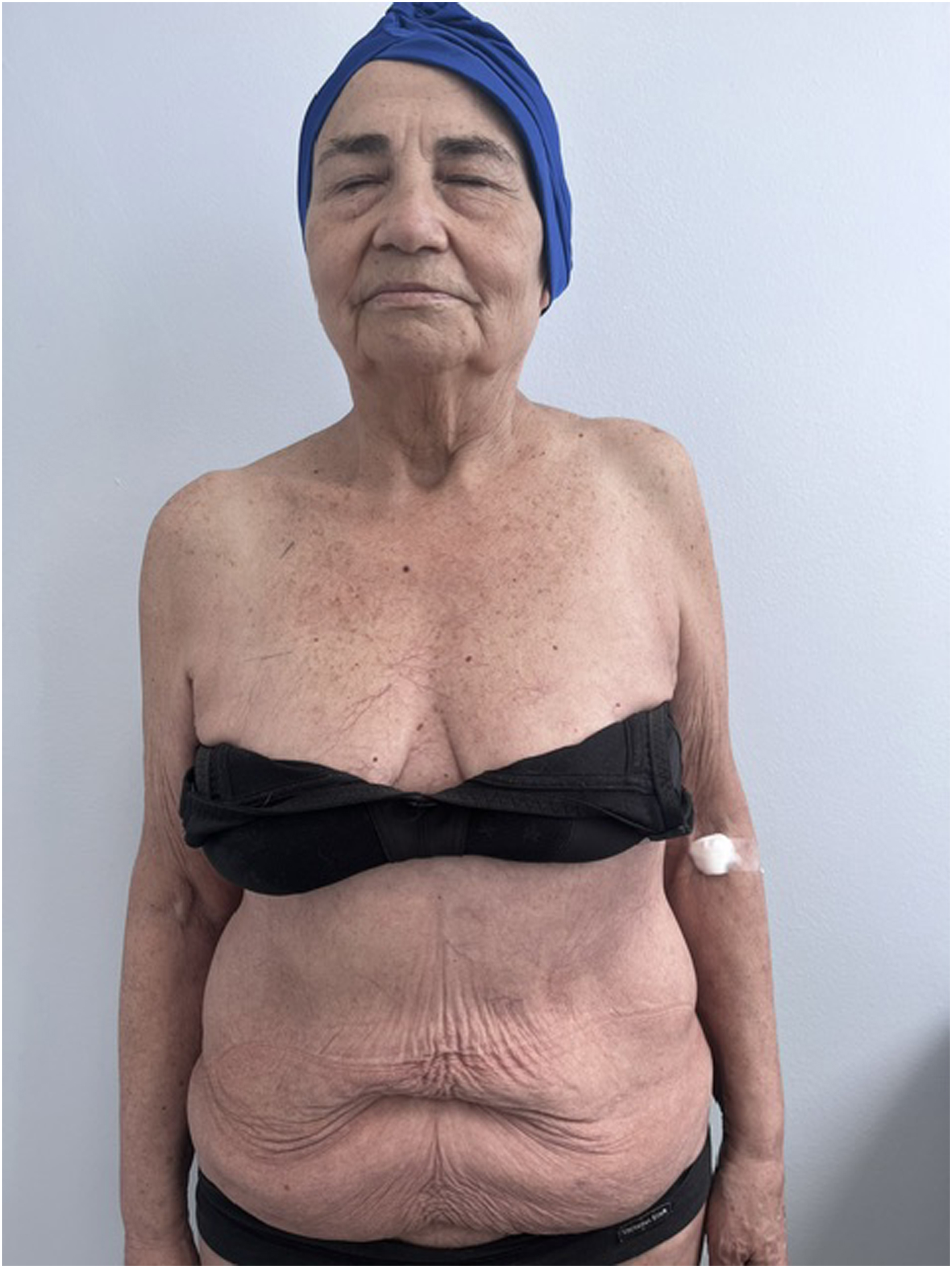

Supplement: Supplementary file 6 [file mmc6.jpg]
